# Neighborhood sociome factors and pediatric asthma exacerbations: Protective role of tree crown density and importance of pharmacy access in Chicago's south side

**DOI:** 10.1111/pai.70127

**Published:** 2025-07-24

**Authors:** Sandra Tilmon, Shashi Bellam, Kathy Bobay, Ellen Cohen, Emily Dillon, Brian Furner, Sarah E. Gray, Julie Johnson, David Meltzer, Doriane Miller, Sharmilee Nyenhuis, Jonathan Ozik, Carlos Santos, Anthony Solomonides, Julian Solway, Elizabeth Zampino, Sanjaya Krishnan, Samuel L. Volchenboum

**Affiliations:** ^1^ Pediatrics University of Chicago Chicago Illinois USA; ^2^ Endeavor Health Research Institute Evanston Illinois USA; ^3^ Loyola University Parkinson School of Health Sciences and Public Health Maywood Illinois USA; ^4^ Life Sciences Carroll University Waukesha Wisconsin USA; ^5^ University of Chicago Medicine Chicago Illinois USA; ^6^ Clinical Research Informatics University of Chicago Chicago Illinois USA; ^7^ Decision and Infrastructure Sciences Division Argonne National Laboratory Lemont Illinois USA; ^8^ Internal Medicine Rush University Medical Center Chicago Illinois USA; ^9^ Computer Science University of Chicago Chicago Illinois USA

**Keywords:** Chicago, health disparities, pediatric asthma, SDOH, social determinants of health, sociome, trees

## Abstract

**Background:**

Pediatric asthma exacerbations remain a critical public health concern, particularly in historically underserved urban settings.

**Objective:**

This study investigates sociome factors—the social context of disease—associated with asthma exacerbations among children living in Chicago's South Side, leveraging clinical and publicly available generalizable census tract‐level datasets from agencies including ChiVes, the City of Chicago Data Portal, EPA, Census Bureau, HUD, NOAA, and more. The aim is to uncover novel hypotheses for potential new interventions.

**Methods:**

A generalized linear model assessed associations with the outcome of asthma exacerbations while accounting for clustering at the patient level. Predictors included all variables from the Sociome Data Commons, including social, environmental, behavioral, economic, housing, and school variables.

**Results:**

Predictors of decreased risk included patient age (+4.8 years, −22%), tree crown density (+6% coverage, −17%), parks per acre (+0.41, −8%), and labor market engagement (+0.8 points, −9%). Conversely, predictors of increased risk included increased distance to the nearest pharmacy (+0.28 miles, +12%), limited English skills (+2.3%, +10%), higher inequality (+0.08 points, +8%), and visits in the Spring (+11%) and Fall (+20%).

**Conclusion:**

The results suggest that tree crown density, a novel finding in the context of asthma exacerbations, may play a protective role. Limited access to health care facilities such as pharmacies continues to complicate care.

**Clinical Implications:**

These findings provide hypotheses for future interventions for long‐standing asthma disparities.

**Abbreviations**
ACS: American Community Survey
AIC: Akaike information criterion
DOT: Department of Transportation
EHR: Electronic health record
EPA: Environmental Protection Agency
FAIR: Findable, Accessible, Interoperable, and Reusable
FEMA: Federal Emergency Management Association
GLM: General linear model
HIPAA: Health Insurance Portability and Accountability Act of 1996
HUD: Housing and Urban Development
ICC: Intraclass correlation coefficient
ICD: International Classification of Diseases
IQR: Interquartile range
IRB: Institutional Review Board
ITM: Institute for Translational Medicine
LASSO: Least absolute shrinkage and selection operator
LOESS: Locally weighted scatterplot smoothing
LR: Likelihood ratio test
NOAA: National Oceanic and Atmospheric Association
PCA: Principal component analysis
PHI: Private health information
QIC: Quasi information criterion
RAPT: Resilience Analysis and Planning Tool
SD: Standard deviation
SDC: Sociome Data Commons

**Key message**

Pediatric asthma in Chicago's South Side has persistent health disparities. The Sociome Data Commons curates novel datasets along with social determinants of health to reflect the full social context of disease and intends to generate new hypotheses for action to reduce health disparities. Predicting asthma exacerbations, findings here reflect predictors of decreased risk of increased tree cover and parks and increased risk from lower health access, poverty‐associated factors, and linguistic isolation.

## 1 INTRODUCTION

Asthma incidence, morbidity, and mortality are deeply influenced by social determinants, including access to health care and neighborhood‐level factors such as pollution.^1^ Such social vulnerabilities are rooted in long‐standing inequities.^2^ Health care in the United States has its own civil rights journey, evolving from the Freedmen's Bureau for previously enslaved individuals into an era of segregated hospitals.^3^ Although formal desegregation ended in 1963,^4^ disparities in health care financing, access, and outcomes remain.^5^ Access itself has been seen to modify asthma treatment effects.^6^

The Institute for Translational Medicine (ITM), a consortium of Chicago‐area research hospitals, launched an initiative aimed at addressing and reversing health disparities. By accounting for the social, environmental, behavioral, psychological, and economic factors that shape health—the full range of lived experience, or the “sociome”—ITM seeks to identify new strategies to reduce health disparities. Further, by intentionally framing health disparities within their historical and social contexts, inadvertent assumptions that such disparities are natural, genetic, or inevitable are avoided. This approach enhances medical and public health research, making it actionable and solution oriented rather than solely descriptive.^7^

Focusing on the sociome is essential because: (1) sociome factors interact with human biology, intensifying or causing disease and injury and (2) illness, regardless of origin, can amplify the adverse effects of these factors.^8^ As part of its research platform, ITM has partnered with Argonne National Laboratory, the University of Chicago's Data Science Institute, and Data for the Common Good to develop the Sociome Data Commons (SDC). This growing resource is designed to collect high‐quality, generalizable public data on sociome factors, following best data practices as outlined in a prior publication.^9^ The SDC brings together social determinants of health data while also integrating novel, carefully curated data, reducing barriers for researchers to incorporate sociome factors into their studies. This in turn supports the discovery of previously unrecognized sociome impacts on health and disease and helps identify potential avenues for novel interventions.

Despite decades of community outreach and interventions by local consortia, Chicago continues to experience persistent disparities in asthma morbidity,^10^, ^11^, ^12^, ^13^ highlighting a need for novel interventions. This paper examines pediatric asthma exacerbations at an urban research hospital serving an under‐resourced area of Chicago. By integrating clinical data with novel datasets from the SDC, we aim to identify factors contributing to acute asthma episodes, offering a foundation for developing new hypotheses and interventions.

## 2 MATERIALS AND METHODS

### 2.1 Clinical data

Data from the University of Chicago Epic (Epic Systems, Verona, WI) electronic health record (EHR) for all pediatric (under 18) visits was collected; this study was approved by the University of Chicago Biological Sciences Division (BSD) IRB #21‐1920, and a waiver of consent was granted. Clinical data (address history, demographics, diagnoses, encounters, labs, medications, and notes) were stored and analyzed on University of Chicago HIPAA‐compliant infrastructure hosted by the Center for Research Informatics. Python^14^ was used for data management.

Encounters were limited to visits for asthma, defined as either an asthma diagnosis^15^ or “asthma” as text in the encounter. Differential diagnoses were present^16^ in 3% of visits and evaluated by a team of specialists (SB, SG, and SN). The National Institute of Health's Value Set Authority Center was our primary source of ICD codes^17^ (Appendix S1: Supplement 1).

An asthma visit for an exacerbation was defined by the specialist team as any of the following:

1. The asthma encounter contains a text description including “exacerbation.”
2. The visit was to the Emergency Department.
3. The patient was prescribed systemic corticosteroids^18^ (Appendix S1: Supplement 1).

This definition ensures that exacerbations are identified comprehensively, capturing both direct documentation of exacerbations and clinical actions likely to be associated with them.

Asthma phenotypes were also defined with the specialist team. Atopic was defined via elevated immunoglobulin E (IgE) or diagnosis of any of rhinitis, eczema, or atopic dermatitis. Other phenotypes included inflammation‐ (T2‐high, elevated eosinophils) or obesity‐related (obesity diagnosis or BMI percentile ≥95%). IgE and eosinophil values came from laboratory results. Asthma control test scores were extracted from the EHR via regular expression and categorized by age but were highly missing.

Missing data were handled in the following ways. All columns with greater than 30% missing were inspected for relevance (e.g., cancer risk among adults); most were found unnecessary and removed. Phenotypes and asthma control were deemed infeasible for imputation due to a lack of antecedent information. Missing data were also examined for patterns: missing insurance (2%) and race/ethnicity (<1%) were not associated with one another by correlation or dendrogram. Filling in missing information backwards and forwards by patient reduced missing insurance by half but had no effect on missing race/ethnicity. Multiple imputation^19^ was conducted for insurance and race/ethnicity, resolving the remaining missing values.

In 2016, visit definitions in the EHR were changed, altering record counts; sensitivity testing was conducted to determine if records before 2017 would be included. Values were tested for statistical significance via chi‐square test for categorical variables and *t*‐tests for numeric variables.

Chicago lies along Lake Michigan to its East and is commonly split into three areas: the South, West, and North sides. Because 97% of asthma patients were from the South Side, analysis was limited to this geography.

Many patients visited the hospital solely for asthma exacerbations; we assumed they received routine care elsewhere and only sought hospital treatment for acute episodes. To avoid bias in our patient sample, we restricted the dataset to only those patients who had at least one routine asthma visit. Before this restriction, asthma exacerbations were 62% of all asthma visits; this fell to 38% after excluding exacerbation‐only patients.

### 2.2 Sociome Data Commons data

In our previous work, we described the development of the SDC, designed according to Findable, Accessible, Interoperable, and Reusable (FAIR) principles, and which included a poverty index derived from the American Community Survey.^9^, ^20^, ^21^ Since then, we have enhanced the commons by curating census‐tract‐level datasets into thematic domains—social, environmental, behavioral, psychological, and economic—to address key challenges such as:

1. Finding relevant datasets.
2. Evaluating data quality and generalizability.
3. Managing, processing, and integrating complex datasets.

These enhancements aim to streamline the integration of sociome factors into research by reducing barriers for researchers.

Additionally, select pollution and housing variables were analyzed for spatial clustering based on the adjacency of census tracts.^22^ Clustering was assessed using Moran's *I*, with emphasis placed on the metric's magnitude and scatterplots of the clusters instead of significant *p*‐values. Only clustering exhibiting strong and consistent patterns in the scatterplots was retained. Raw data and Python code are included in the SDC to ensure transparency to users.

In this paper, we further refined SDC data. Threshold effects identified through partial dependence plots led to the conversion of facility counts, such as community centers and landfills, into binary indicators of presence within census tracts.

### 2.3 Analytic dataset

Geocoding to latitude, longitude, and census tract was conducted with GoogleV3 and GeoPy^23^ for SDC public dataset addresses; this method was chosen for ease of use given no need to protect protected health information (PHI). To maintain security and privacy of PHI, the HIPAA‐compliant DeGAUSS platform was used; patient address history was available for visit‐specific addresses and geocoded to latitude, longitude, census block, and census tract.^24^ For interpretability, distance in miles between patient location and points of interest was calculated via the Haversine formula.^25^ Computation time was reduced by using spatial indices and parallel processing. Points of interest included the closest health care site (primary care, hospital, and pharmacy) and various pollution sites.^26^, ^27^

To prevent extreme values from influencing the analysis, continuous variables were identified as outliers by Tukey's 1.5 * the interquartile range (IQR) rule.^28^ The IQR, defined as the 25th to 75th percentiles, represents the middle 50% of the data. With this method, outliers are values outside the lower limit (quartile 1–1.5 * IQR) or upper limit (quartile 3 + 1.5 * IQR). Rather than excluding these outliers, any data points beyond these thresholds were capped at the boundary values. This approach minimizes the impact of extreme values while retaining the full dataset for analysis.

Given seasonal effects with exacerbations, visits were categorized by quarter of the year. Summer (quarter 3) was used as the referent season and excluded from the analytic dataset.

Clinical and SDC data were joined at the patient's census tract. Weather metrics (temperature and humidity) with changes from the previous day were joined at the visit date.^29^ After making binary indicators for categorical variables, the final dataset had 199 variables, 35 of which were patient‐specific and the remainder describing the census tract. Excluding binary variables, continuous variables were rescaled to mean 0 and standard deviation (SD) 1 with standard scaler.^30^

### 2.4 Variable selection

Variable selection was performed using R^31^ by combining variables chosen through three disparate methods: stepwise selection, least absolute shrinkage and selection operator (LASSO), and principal component analysis (PCA). Stepwise selection was applied in two ways: forward (starting with an empty model and adding variables) and backward (starting with a full model and removing variables). LASSO is a regularization method that penalizes variables by shrinking less important variables' coefficients to 0.

Unlike stepwise selection or LASSO, PCA does not predict an outcome. Instead, it combines variables into a smaller set of components, with each variable's weight reflecting its importance, thus ranking variables. For PCA, variables in the first component with loadings above the median were retained. Variables from all methods were combined, and highly correlated variable pairs (|0.8| or greater) were reduced to a single representative variable (Appendix S1: Supplement 2).

Asthma visits were modeled with an exacerbation as the outcome. Patient race/ethnicity and insurance variables were excluded to prevent overfitting on race, because public health insurance is a known proxy for poverty, and because of the homogeneity of both race/ethnicity (84% non‐Hispanic Black) and insurance (65% Medicare or Medicaid).

### 2.5 Modeling

A multi‐level generalized linear model (GLM) was conducted using STATA 18.^32^ GLMs are semi‐parametric and account for clustering at the patient level while also allowing for variability in both the number and timing of repeat visits.^33^ The intraclass correlation coefficient (ICC) estimates how much variance is accounted for by the multilevel factor^33^ and was calculated separately for the patient, the patient's census tract, and a combination of both.

All parameters were tested. A logit or probit link was chosen via a likelihood ratio (LR) test. Different correlation structures (independent, exchangeable, and auto‐regressive (1)) were tested via quasi information criterion (QIC).^34^

A “saturated” model was run, including all 129 variables from the variable selection as a preliminary model. To balance model complexity with predictive performance, this model was compared to a reduced version limited to predictors with an upper *p*‐value threshold of .10. This reduced model is nested within the saturated model, enabling the use of a LR test to evaluate the fit between them. The null hypothesis is that the reduced model performs as well as the saturated (e.g., *p* ≥ .05), while the alternative hypothesis is that they perform differently (e.g., *p* < .05). Akaike information criterion (AIC) values, which assess model fit while accounting for the number of variables, were also compared,^34^ with lower AIC values indicating better fit.

To examine overall explanatory power, McFadden's Pseudo R‐Squared was calculated as 1 – (log likelihood of final model/log likelihood of null model).^35^ A null model is a baseline model that contains the outcome, but no predictors.

## 3 RESULTS

### 3.1 EHR artifact at the end of 2016: Sensitivity check

The sensitivity check for the EHR artifact showed significant and meaningful differences in insurance types. However, the plan was to exclude insurance, as the majority of patients have public insurance, mostly Medicare with some Medicaid, and this is a known proxy for poverty.

Statistically significant differences were also found with asthma phenotypes. However, these variables were too sparse to include in the model (Appendix S1: Supplement 3).

### 3.2 Descriptives

The pediatric asthma patient population mainly consisted of non‐Hispanic Black children (84%), covered by public insurance (65%), with an average age of 8 years old, and a slight male predominance (58%). Residential mobility was small, with most residing in a single census tract during the study period. On average, patients had 4 visits, though visit frequency varied widely with a SD of 5 visits (Table 1).

**TABLE 1:** Descriptive characteristics of pediatric patients with at least one routine visit for asthma by ever having a visit for an asthma exacerbation.

	Overall	Ever had an exacerbation	Never had an exacerbation
Totals	3421	1585	1836
Male, *n* (%)	1970 (58%)	907 (57%)	1063 (58%)
Age mean (SD)	8.3 (5.1)	7.8 (4.8)	8.7 (5.3)
Race/ethnicity, *n* (%)			
Hispanic	241 (7%)	92 (6%)	149 (8%)
Non‐Hispanic			
Asian or Middle Eastern	50 (2%)	19 (1%)	31 (2%)
Black/African‐American	2887 (84%)	1385 (87%)	1502 (82%)
White	198 (6%)	66 (4%)	132 (7%)
Multiple and all others	45 (1%)	23 (1%)	22 (1%)
Insurance, *n* (%)			
Private	1139 (33%)	465 (29%)	674 (37%)
Private with Medicaid	40 (1%)	20 (1%)	20 (1%)
Public: Medicare, Medicaid	2219 (65%)	1089 (69%)	1130 (62%)
Self pay and other	23 (<1%)	11 (<1%)	12 (<1%)
Number of visits, mean (SD)	4.1 (5.1)	5.1 (6.7)	6.7 (6.4)
Number of census tracts, mean (SD)	1.3 (0.6)	0.6 (1.5)	1.5 (0.8)

Abbreviations: SD: Standard deviation.

Most patients had not experienced exacerbations. Patients without exacerbations tended to be slightly younger (7.8 years vs. 8.7 years), had fewer visits (5.1 vs. 6.7), and resided in fewer home census tracts (0.6 vs. 1.5). Race‐ and insurance‐based differences were present: non‐Hispanic Black patients were slightly more likely to experience exacerbations (87% to 82%) compared to non‐Hispanic White patients (4% vs. 7%). Similarly, patients with public insurance were slightly more likely to have had exacerbation visits (69% vs. 62%) compared to those with private insurance (29% to 37%) (Table 1).

### 3.3 Variable selection

When comparing methods, 37 variables were selected by all 3 methods. Thirty‐three variables were selected by both stepwise selection and LASSO, 27 by LASSO and PCA, and only 1 by stepwise and PCA. For variables not detected by either other method, stepwise selected 2, PCA selected 5, and LASSO selected 37.

From the original 199 candidate variables, 88 were selected from stepwise selection, 152 from LASSO, and 84 from PCA. After removing 23 highly correlated variables, the final count was 129 (Appendix S1: Supplement 2).

### 3.4 Modeling

GLM ICC values were as follows: for patients, 27% of variance; census tract, 6%; both, 27%. Given the negligible amount of increase in variance explained from census tracts, the model included patients only as the multiple‐level variable.

**FIGURE 1:**
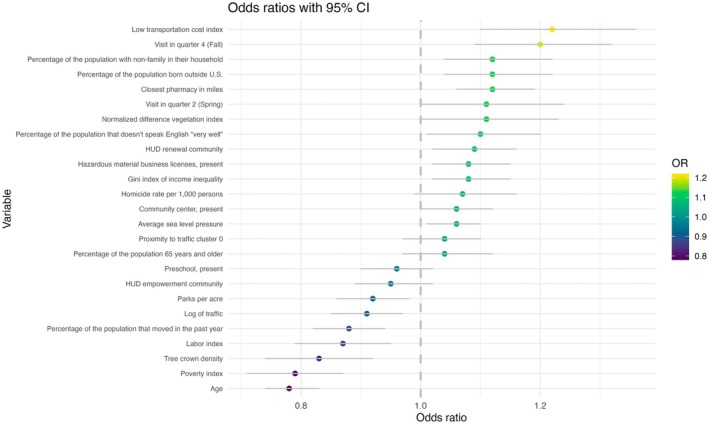
Odds ratios and 95% confidence intervals, sorted descending by odds ratio. The dashed line at 1.0 marks no association with the outcome; any variable with a confidence interval crossing 1 is not statistically significant.

After filtering the saturated model to variables with *p*‐values ≤.10, the variable count was reduced from 129 to 25 (Table 2 and Figure 1). The reduced model did not differ in explanatory power from the saturated one and so was selected (*χ* ^2^_(101)_ = 79.05, *p* = .9480). The AIC for the reduced model (16,750.82) was also lower than for the saturated one (16,873.78), showing a better fit. The final model, however, had a notably low McFadden's pseudo *r* ^2^ of .02.

**TABLE 2:** Final model odds ratios, 95% confidence intervals, data sources, and selection method.

Category	Variable	Data source	Final model OR for an exacerbation (95% CI)	Final model *p*‐value	Method(s) which selected the variable
*Patient‐specific variables*					
	Age	Electronic health record (EHR)	0.78 (0.74, 0.83)	<.001	LASSO, Stepwise
	Closest pharmacy in miles (closest_pharma_miles)	EHR, Chicago Data Portal^36^	1.12 (1.06, 1.19)	<.001	All
*Patient‐specific visit date variables*					
	Average sea level pressure (avgsealvlpress)	National Oceanic and Atmospheric Association (NOAA)^29^	1.06 (1.01, 1.10)	.008	Stepwise
	Visit in quarter 2 (visitq2)	EHR	1.11 (1.00, 1.24)	.042	LASSO, Stepwise
	Visit in quarter 4 (visitq4)	EHR	1.20 (1.09, 1.32)	<.001	LASSO, Stepwise
*Neighborhood (census tract) variables*					
	Community center, present (commctrcount_yn)	Chicago Data Portal	1.06 (1.00, 1.12)	.039	LASSO, Stepwise
	Gini index of income inequality (gini): 0 as perfect equality, 1 as perfect inequality	Federal Emergency Management Association (FEMA), Resilience Analysis and Planning Tool (RAPT)^37^	1.08 (1.02, 1.15)	.014	LASSO, PCA
	Hazardous material business licenses, present (hazardcount_yn)	Chicago Data Portal^36^	1.08 (1.02, 1.15)	.009	LASSO, Stepwise
	Homicide rate per 1000 persons (homiciderate1000)	Chicago Data Portal,^36^ American Community Survey (ACS)^21^	1.07 (0.99, 1.16)	.093	LASSO, Stepwise
	HUD empowerment zone (hudempowerzone)	Housing and Urban Development (HUD)^38^	0.95 (0.89, 1.02)	.143	LASSO, PCA
	HUD renewal community (hudrenewalcomm)	HUD^38^	1.09 (1.02, 1.16)	.010	LASSO, Stepwise
	Labor market engagement index (lbr_idx) higher value, higher engagement	HUD^39^	0.87 (0.79, 0.95)	.003	LASSO, Stepwise
	Low transportation cost index (tcost_idx) higher value, lower cost	Department of Transportation (DOT)^40^	1.22 (1.10, 1.36)	<.001	All
	Normalized difference vegetation index (ndvi)	ChiVes^41^	1.11 (1.00, 1.23)	.042	All
	Parks per census tract acre (parks_per_acre)	Chicago Data Portal,^36^ ACS^21^	0.92 (0.86, 0.98)	.006	LASSO, PCA
	Percentage of all ACS occupied housing units where a householder lives alone or with nonrelatives only; includes unmarried same‐sex couples where no relatives of the householder are present (pct_nonfamily_hhd_acs)	ACS^21^	1.12 (1.04, 1.22)	.005	LASSO, PCA
	Percentage of all ACS occupied housing units where no one ages 14 years and over speaks English only or speaks English “very well” (pct_eng_vw_acs)	ACS^21^	1.1 (1.01, 1.2)	.028	LASSO
	Percentage of the ACS population who were not a citizen of the United States at birth. This includes respondents who indicated that they were a US citizen by naturalization or not a US citizen. (pct_born_foreign_acs)	ACS^21^	1.12 (1.04, 1.22)	.005	LASSO
	Percentage of the ACS population aged 1 year and over that moved from another residence in the United States or Puerto Rico within the last year (pct_diff_hu_1yr_ago_acs)	ACS^21^	0.88 (0.82, 0.94)	<.001	All
	Percentage of the ACS population that is 65 years old or over (pct_pop_65plus_acs)	ACS^21^	1.04 (0.97, 1.12)	.298	LASSO, PCA
	Poverty index (pca1): Higher values represent higher poverty	ACS^21^	0.79 (0.71, 0.87)	<.001	All
	Preschool, present (preschn_yn)	Chicago Data Portal^36^	0.96 (0.9, 1.02)	.162	LASSO, Stepwise
	Proximity to traffic cluster 0 (ptraf_4_clus_00)	Spatial clustering of EPA environmental justice screen data^42^	1.04 (0.97, 1.10)	.264	LASSO, Stepwise
	Traffic, log‐transformed (logtraf)	ChiVes^41^	0.91 (0.85, 0.97)	.006	All
	Tree crown density (trees_crown_den)	ChiVes^41^	0.83 (0.74, 0.92)	.001	All

For variable selection, the most successful selection came from LASSO, with 19 of the 20 significant variables in the final model. In contrast, stepwise selection and PCA resulted in 14 and 10 variables, respectively. For patient‐specific factors, higher age predicted decreased risk, with a one‐SD increase reducing exacerbation odds (4.8 years, −22%). In contrast, odds increased with visits during Spring (quarter 2, +11%) and Fall (quarter 4, +20%). Among continuous variables, increased odds occurred with 1 SD increases in distance to the closest pharmacy (0.28 miles, +12%) and average sea level pressure (0.21 millibars, indicating weather changes, +6%).

For census tract‐level factors, several predictors of decreased risk were observed, described as the reduction in exacerbation odds per 1 SD increase: poverty index (3.1 points, −21%), tree crown density (6.4%, −17%), labor market engagement index (16.2 points, −13%), recent movers (7.8%, −12%), log‐transformed traffic (0.8, −9%), and parks per acre (0.41, −8%).

Conversely, predictors of increased risk were linked to other census tract factors: the presence of a community center (+6%), hazardous material business licenses (+8%), and designation as a HUD renewal community (+9%). Among continuous factors, a 1 SD increase raised odds as follows: GINI coefficient of inequality (0.08, +8%), low transportation cost index (6.4 points, +13%), NDVI (6 points, +11%), non‐family household percentage (13.6%, +12%), limited English proficiency percentage (2.3%, +10%), and foreign‐born population percentage (7.7%, +12%) (Table 2).

The final variables were examined with a correlation matrix (Appendix S1: Supplement 4). While highly correlated variables had been excluded during data pre‐processing, several moderate correlations remained:

- The labor index and the poverty index (−0.73).
- The homicide rate with the labor index (−0.63) and the poverty index (0.67).
- NDVI and tree crown density (0.77).
- The percentage of the population born outside the United States and lower English language skills (0.77).
- The transportation index with both the percentage of households that moved within the last year (0.53) and the percentage of non‐family households (0.58).

Among the above, all but the homicide rate were significant in the final model.

**FIGURE 2:**
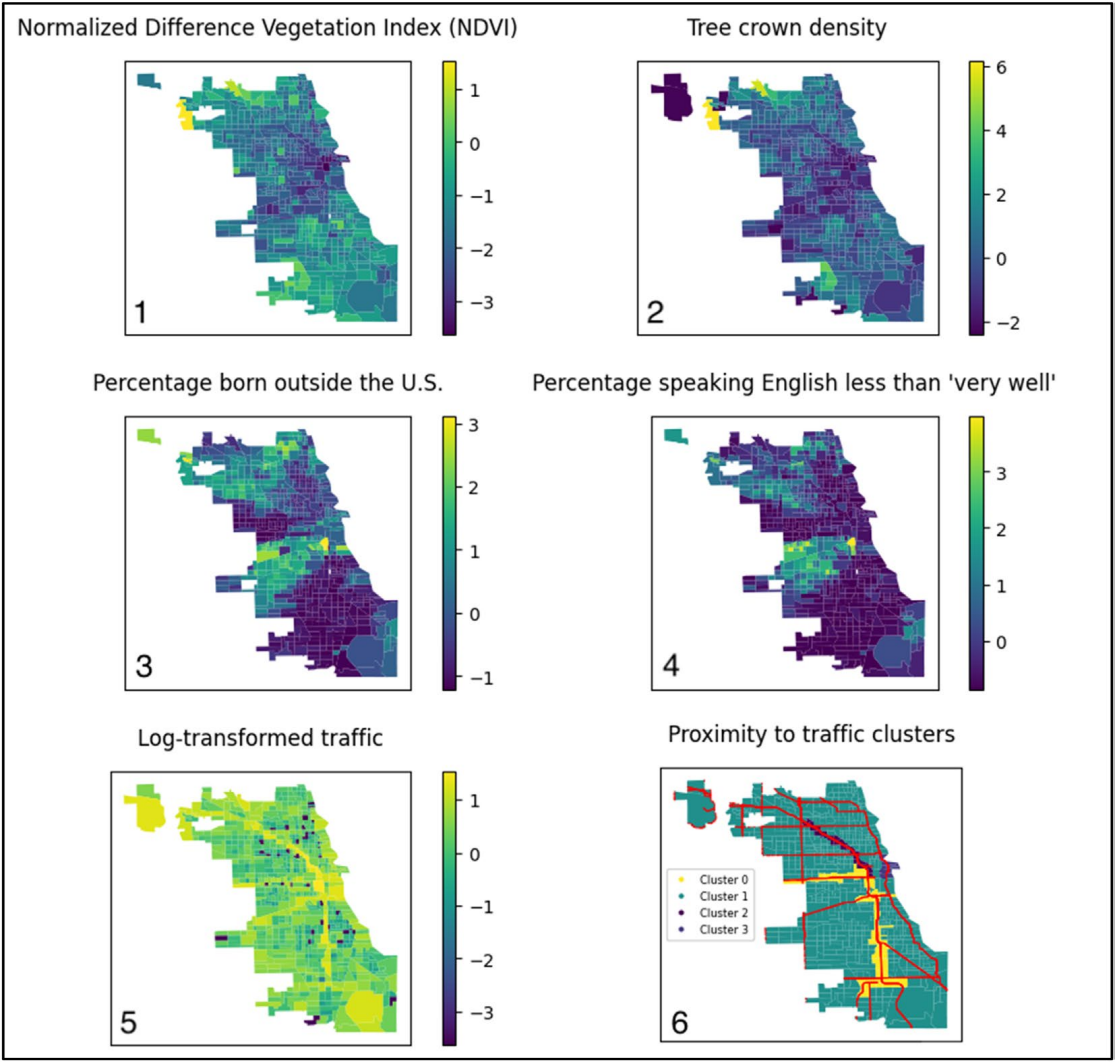
Six maps of Chicago census tracts, where each row shows two correlated variables. Data for maps 1–5 have been scaled to mean 0 and standard deviation 1. Map 1: Normalized Difference Vegetation Index. Map 2: Tree crown density. Map 3: Percentage of the population born outside the United States. Map 4: Percentage speaking English less than “very well.” Map 5: Log‐transformed traffic. Map 6: Clusters of “proximity to traffic” with major highways in red.

The NDVI reflects green space, with both NDVI and tree crown density showing the lowest levels in a diagonal area in the center of the city. The percentage of people born outside the United States overlaps with limited English proficiency. Log‐transformed traffic is highest along highways, visible in the proximity to traffic clusters map (in red) (Figure 2).

**FIGURE 3:**
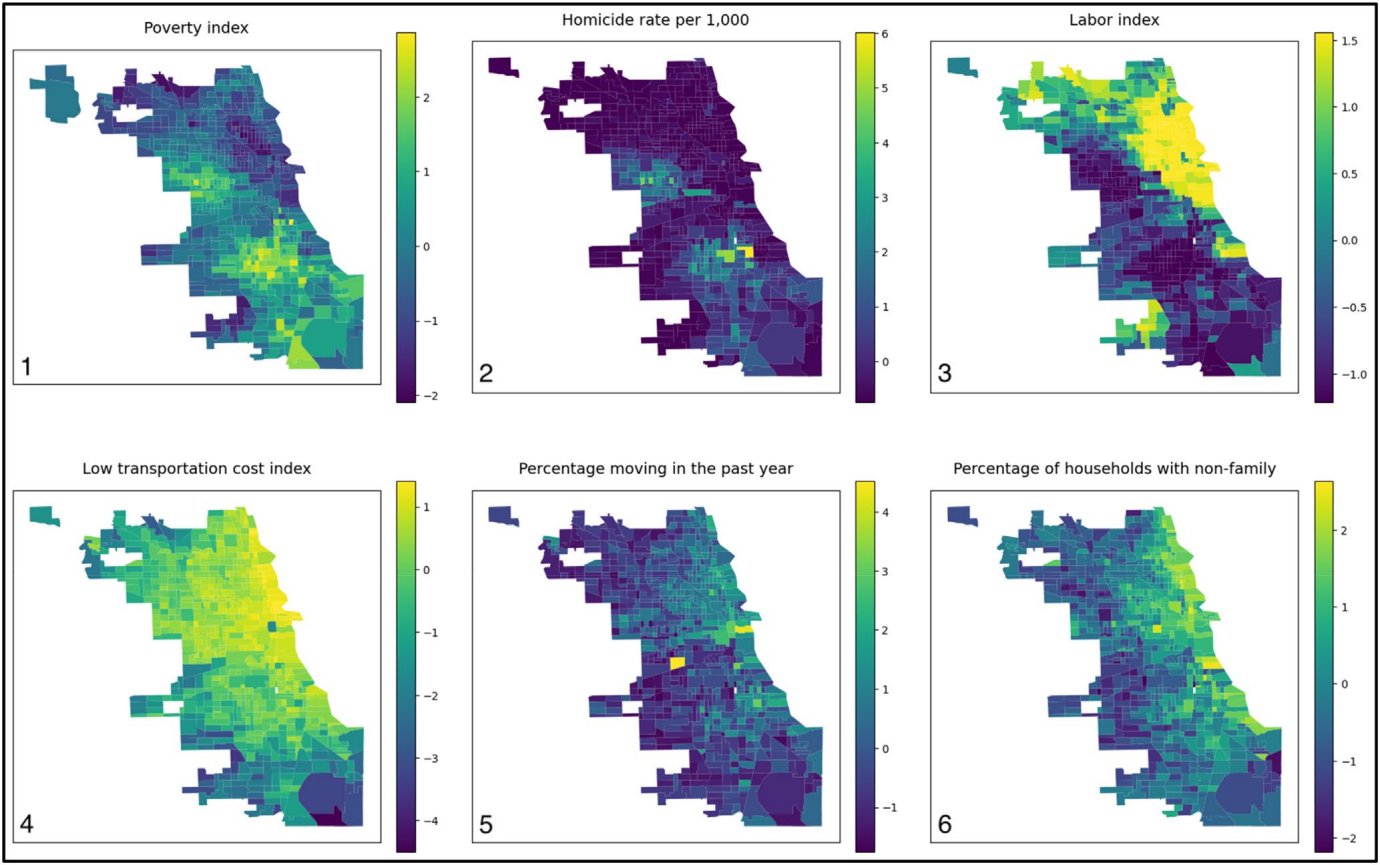
6 maps of Chicago census tracts where each row shows 3 correlated variables. All map data has been scaled to mean 0 and standard deviation 1. Map 1: Poverty index from the American Community Survey; Map 2: Homicide rate per 1000 persons; Map 3: Labor index; Map 4: Low transportation cost index; Map 5: Percentage of households moving in the past year; Map 6: Percentage of households with non‐family.

The poverty index reveals the highest poverty on the South Side of Chicago and the West‐Northwest. The homicide rate per 1000 persons closely mirrors the poverty index, while labor market engagement contrasts with both, highest in the Northeast. The low transportation cost index is also highest in the Northeast but extends further into the Northwest. The percentages of people moving in the past year and households with non‐family are both highest in the East, where Chicago borders Lake Michigan (Figure 3).

**FIGURE 4:**
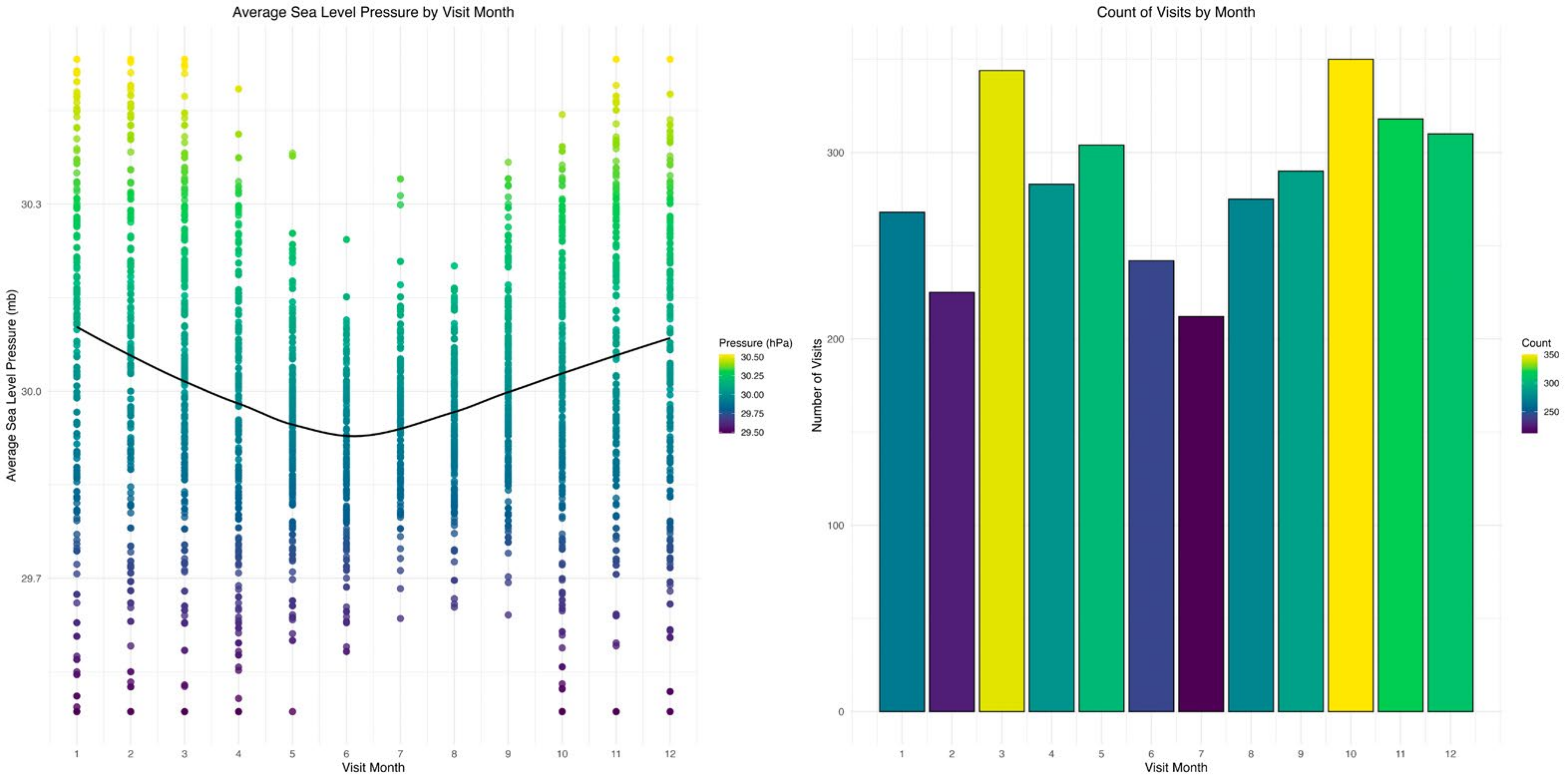
Seasonality: Average sea level pressure by visit month (left) and visits by month (right), both with locally weighted scatterplot smoothing (loess) trend lines in black.

Average sea level pressure changes with the seasons, with both lower averages and variability during the middle of the year—the summer months. This is reflected in reduced asthma visits during the same season (Figure 4).

## 4 DISCUSSION

### 4.1 Summary of findings

This study leveraged the Sociome Data Commons to generate hypotheses on sociome factors influencing asthma exacerbations. We identified several predictors of increased and decreased risk, underscoring the complex interplay between environmental, socioeconomic, and healthcare‐related factors. Increasing patient age was associated with a lower risk of exacerbations, while greater distance to the nearest pharmacy suggested reduced access to care. Seasonal variation played a role, with exacerbations peaking in the Spring and Fall, possibly due to increased allergen exposure, viral circulation, and crowding.

Tree crown density emerged as a novel predictor of reduced risk, marking the first instance (to our knowledge) of an association between tree coverage and lower asthma exacerbations. Other protective factors included green space, measured by parks per acre, and higher labor market engagement, which may reflect environmental and socioeconomic conditions that support better respiratory health.

Conversely, predictors of increased risk often align with markers of socioeconomic disadvantage, including higher income inequality (GINI values), HUD renewal community status, and lower English proficiency—suggesting potential influences of poverty, social isolation, and linguistic barriers.

### 4.2 Findings in context of other studies and potential applications

The relationship between urban greenery and asthma outcomes has been inconsistently reported in prior literature. While some studies have suggested that tree coverage may exacerbate respiratory conditions due to allergen production,^44^, ^45^ our findings align with emerging research supporting the beneficial effects of urban trees on air quality and respiratory health.^46^, ^47^ This supports calls for further investigation into the role of tree density in mitigating asthma risk and suggests that increasing urban tree cover could serve as a community‐level intervention. Notably, the city of Chicago launched a tree equity plan in 2022,^48^ highlighting the growing recognition of urban forestry as a potential public health strategy. However, given the time required for trees to mature and impact air quality, sustained investment is essential.

Similarly, the association between green space and respiratory health aligns with prior research suggesting that access to parks may promote physical activity, reduce stress, and improve air quality. Socioeconomic factors such as higher labor market engagement may indicate improved economic stability, healthcare access, and environmental conditions that collectively reduce asthma exacerbation risk.

Conversely, neighborhood‐level factors associated with increased risk—such as income inequality and linguistic isolation—reinforce existing evidence on the social determinants of health. These findings emphasize the need for policies that address structural inequities affecting asthma morbidity. However, some associations, such as the counterintuitive negative relationship between traffic levels and asthma risk, warrant further exploration. These may reflect residual confounding, geographic dependencies, or model adjustments for spatial clustering.

### 4.3 Limitations

Several limitations should be acknowledged. The study was restricted to a geographically homogeneous patient population within a pediatric asthma service area, limiting generalizability. Ongoing efforts to integrate data from multiple hospitals through ITM will help validate and extend these findings. Additionally, the STATA GLM^32^ package allowed for only a single autoregressive lag, and while the model accounted for patient‐level clustering, future work should explore alternative longitudinal structures. While further experimentation on tree cover as an intervention is encouraged, tree type and pollen count, data not available here, should be included. Finally, potential ecological fallacies and mismatches between census tract and individual‐level data highlight the need for cautious interpretation and further investigation using finer‐scale datasets.^43^

## 5 CONCLUSION

Pediatric asthma health disparities in Chicago reflect deeply entrenched systemic inequities. Many of the identified risk factors—such as tree crown density and pharmacy access—highlight the uneven distribution of environmental and healthcare resources. Addressing these disparities requires multifaceted interventions integrating medical, public health, and policy approaches. Expanding tree equity initiatives, improving healthcare accessibility, and addressing social determinants of health could play a crucial role in reducing asthma morbidity. Further research is necessary to confirm these associations and explore targeted interventions in collaboration with broader policy efforts aimed at systemic change.

## AUTHOR CONTRIBUTIONS

**Sandra Tilmon:** Methodology; conceptualization; software; data curation; investigation; validation; formal analysis; writing – original draft; writing – review and editing; visualization. **Shashi Bellam:** Investigation; writing – review and editing. **Kathy Bobay:** Writing – review and editing. **Ellen Cohen:** Conceptualization; supervision; funding acquisition; writing – review and editing. **Emily Dillon:** Writing – review and editing; conceptualization. **Brian Furner:** Conceptualization; software; supervision. **Sarah E. Gray:** Investigation; writing – review and editing. **Julie Johnson:** Methodology; resources; data curation. **David Meltzer:** Writing – review and editing. **Doriane Miller:** Writing – review and editing. **Sharmilee Nyenhuis:** Conceptualization; investigation; writing – review and editing. **Jonathan Ozik:** Conceptualization; writing – review and editing. **Carlos Santos:** Conceptualization; investigation; writing – review and editing. **Anthony Solomonides:** Conceptualization; writing – review and editing. **Julian Solway:** Conceptualization; investigation; writing – review and editing; funding acquisition. **Elizabeth Zampino:** Project administration. **Sanjaya Krishnan:** Methodology; software; conceptualization; supervision; funding acquisition; writing – review and editing. **Samuel L. Volchenboum:** Conceptualization; writing – review and editing; funding acquisition.

## FUNDING INFORMATION

NIH UL1TR002389‐07.

## CONFLICT OF INTEREST STATEMENT

S.B. served on an advisory board for Sanofi. E.F.D. receives consulting fees from Argus Cognitive for software medical devices. S.E.G. served on an advisory board for Sanofi. S.N. served on an advisory board for Avillion/Astra Zeneca, receives royalties from Wolters‐Kluwer and Springer, and research funding from NIH and Asthma Allergy Foundation of America. A.S. Holds voluntary positions in the American Medical Informatics Association and is an equity investor in healthcare companies and other industries. C.S. served on advisory boards for Gilead and Merck, receives royalties from Wolters‐Kluwer, and receives research funding from CDC. J.S. reports a potential financial interest in PulmOne Advanced Medical Devices, Ltd., Israel, and research grant funding from NIH, NSF, and the Respiratory Health Association of Metropolitan Chicago. S.T. receives consulting fees from Aetna Medicare. S.L.V. is co‐founder and Chief Medical Officer for Litmus Health, Inc., and receives consulting royalties from CVS Accordant.

## PEER REVIEW

The peer review history for this article is available at https://www.webofscience.com/api/gateway/wos/peer‐review/10.1111/pai.70127.

## Supporting information


Appendix S1.
