# Supplementary material for: Neighborhood sociome factors and pediatric asthma exacerbations: Protective role of tree crown density and importance of pharmacy access in Chicago's south side
Source: Pediatr Allergy Immunol. 2025 Jul 24;36(7):e70127. doi: 10.1111/pai.70127 (PMC12290247; doi:10.1111/pai.70127)
Supplement: Supplementary file 1 — Appendix S1. [file PAI-36-e70127-s001.docx]

## Supplement 1: Definitions

### ICD codes used

| Concept and NIH object identifier (OID) or citation | Codes |
| --- | --- |
| Asthma ICD9CM OID 2.16.840.1.113883.3.117.1.7.1.904 | ['493.00', '493.01', '493.02', '493.10', '493.11', '493.12', '493.81', '493.82', '493.90', '493.91', '493.92'] |
| Asthma OID 2.16.840.1.113762.1.4.1106.60 | ['J45', 'J45.2', 'J45.20', 'J45.21', 'J45.22', 'J45.3', 'J45.30', 'J45.31', 'J45.32', 'J45.4', 'J45.40', 'J45.41', 'J45.42', 'J45.5', 'J45.50', 'J45.51', 'J45.52', 'J45.9', 'J45.90', 'J45.901', 'J45.902', 'J45.909', 'J45.99', 'J45.991', 'J45.998'] |
| Differential diagnoses. Johnson, J., Abraham, T., Sandhu, M., Jhaveri, D., Hostoffer, R., Sher, T. (2019). Differential Diagnosis of Asthma. In: Allergy and Asthma. Springer, Cham. https://doi.org/10.1007/978-3-030-05147-1_17 | ['J44.0', 'J44.1', 'J44.9', 'K21.9', 'K21.01', 'J01.90', 'J01.91', 'J32.0', 'J32.1', 'J32.2', 'J32.3', 'J32.4', 'J32.8', 'J32.9', 'I50.814', 'I50.9', 'J38.3', 'T78', 'J70.8', 'C80.1', 'D86.0', 'D86.2', 'D86.9', 'J67.0', 'J67.1', 'J67.2', 'J67.3', 'J67.4', 'J67.5', 'J67.6', 'J67.7', 'J67.8', 'J67.9', 'I27.0', 'I27.20', 'I27.21', 'I27.22', 'I27.23', 'I27.24', 'I27.29', 'J98.01', 'J84.81', 'E84.9', 'J82.89', 'M30.1', 'M31.3', 'M31.7'] |
| Atopic Dermatitis ICD10CM OID 2.16.840.1.113762.1.4.1078.158 | ['L20.0', 'L20.81', 'L20.82', 'L20.83', 'L20.84', 'L20.89', 'L20.9'] |
| Atopy ICD 9 | ['691.8', 'V15.09', '477.9', 'V13.3', '684', '692.9', 'V15.09', '493.00', '493.01'] |
| Atopy ICD 10 | ['L20.9', 'L20.84', 'L20.83', 'L20.9', 'Z88.9', 'L20.89', 'J30.9', 'Z87.2', 'L23.9', 'J45.902', 'L20.81', 'J45.909'] |
| Conditions related to asthma ICD10CM OID 2.16.840.1.113762.1.4.1078.622 | ['E66.01', 'E66.09', 'E66.1', 'E66.2', 'E66.3', 'E66.8', 'E66.9', 'G47.33', 'J01.00', 'J01.01', 'J01.10', 'J01.11', 'J01.20', 'J01.21', 'J01.30', 'J01.31', 'J01.40', 'J01.41', 'J01.80', 'J01.81', 'J01.90', 'J01.91', 'J06.0', 'J06.9', 'J30.0', 'J30.1', 'J30.2', 'J30.5', 'J30.81', 'J30.89', 'J30.9', 'J31.0', 'J31.1', 'J31.2', 'J40', 'J41.0', 'J41.1', 'J41.8', 'J43.0', 'J43.1', 'J43.2', 'J43.8',  'J43.9', 'J44.0', 'J44.1', 'J44.81', 'J44.89', 'J44.9', 'J47.0', 'J47.1', 'J47.9', 'K21.9'] |
| Obesity and overweight | ['E66.01', 'E66.09', 'E66.1', 'E66.2', 'E66.3', 'E66.8', 'E66.9'] |
| Apnea | ['G47.33'] |
| Sinusitis | ['J01.00', 'J01.01', 'J01.10', 'J01.11', 'J01.20', 'J01.21', 'J01.30', 'J01.31', 'J01.40', 'J01.41', 'J01.80', 'J01.81', 'J01.90', 'J01.91'] |
| Upper respiratory and laryngopharyngitis | ['J06.0', 'J06.9'] |
| Rhinitis | ['J30.0', 'J30.1', 'J30.2', 'J30.5', 'J30.81', 'J30.89', 'J30.9', 'J31.0', 'J31.1', 'J31.2'] |
| Bronchitis | ['J40', 'J41.0', 'J41.1', 'J41.8'] |
| Emphysema | ['J43.0', 'J43.1', 'J43.2', 'J43.8', 'J43.9'] |
| Chronic Obstructive Pulmonary Disease (COPD) | ['J44.0', 'J44.1', 'J44.81', 'J44.89', 'J44.9'] |
| Bronchiectasis | ['J47.0', 'J47.1', 'J47.9'] |

Table 1.1: ICD Codes used

###

### Medications

| Concept | Medication names or NIH object identifier (OID) |
| --- | --- |
| Recent therapies | Omalizumab, dupilumab, mepolizumab, reslizumab, benralizumab |
| Corticosteroids | OID 2.16.840.1.113883.3.464.1003.196.11.1483 |
| Corticosteroids, Systemic | OID 2.16.840.1.113883.3.3616.200.110.102.2061 |

Table 1.2: Medications used

##

## Supplement 2: Highly correlated variables


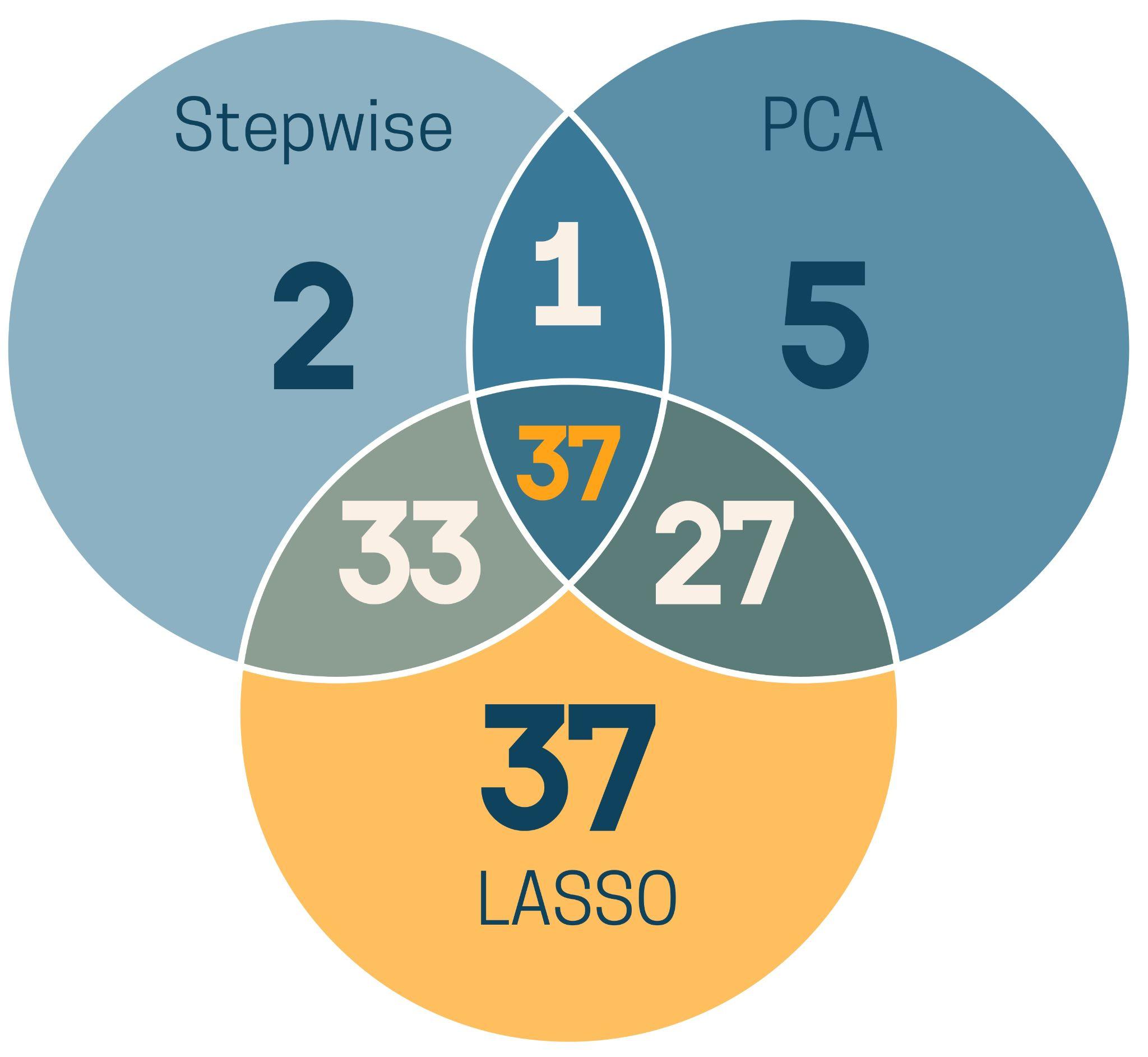


**Figure 2.1**: Variable selection. Variables selected by any of three methods: forwards and backwards stepwise selection, principal component analysis (PCA), and least absolute shrinkage and selection operator (LASSO). The figure shows the variables selected by each method alone and the common variables selected by multiple methods.

| Selected variable | Removed variable | Correlation |
| --- | --- | --- |
| AvgFamily_case_management | AvgPublic_health_nursing | 0.91 |
| AvgDewPtTemp | AvgWetBulbTemp | 0.99 |
| AvgDewPtTemp_change | AvgWetBulbTemp_change | 0.94 |
| AvgDryBulbTemp_change | AvgWetBulbTemp_change | 0.92 |
| closest_air_rls_miles | closest_asphalt_miles | -0.84 |
| closest_air_rls_miles | closest_dist_ctr_miles | 0.96 |
|  | closest_asphalt_miles, closest_dist_ctr_miles | -0.84 |
| closest_air_rls_miles | closest_epa_super_miles | -0.84 |
|  | closest_asphalt_miles, closest_epa_super_miles | 1.00 |
|  | closest_dist_ctr_miles, closest_epa_super_miles | -0.84 |
| heatisl | heat_5q_clus_0.0 | -0.80 |
| LILATracts_Vehicle | HUNVFlag | 0.95 |
| ndvi_4_clus_1.0 | ndvi_4_clus_3.0 | -0.93 |
| closest_air_rls_miles | OZONE | 0.86 |
|  | closest_dist_ctr_miles, OZONE | 0.85 |
| pct_NonFamily_HHD_ACS | pct_Rel_Family_HHD_ACS | -1.00 |
| pct_NonFamily_HHD_ACS | pct_Sngl_Prns_HHD_ACS | 0.96 |
|  | pct_Rel_Family_HHD_ACS, pct_Sngl_Prns_HHD_ACS | -0.96 |
| pnlp_3_clus_0.0 | pnlp_3_clus_1.0 | -0.99 |
| closest_air_rls_miles | PNPL | 0.92 |
|  | closest_asphalt_miles, PNPL | -0.91 |
|  | closest_dist_ctr_miles, PNPL | 0.90 |
|  | closest_epa_super_miles, PNPL | -0.91 |
| pnlp_3_clus_0.0 | PNPL | -0.85 |
|  | pnlp_3_clus_1.0, PNPL | 0.83 |
| prmp_6_clus_2.0 | PRMP | -0.84 |
| ptraf_4_clus_0.0 | PTRAF | 0.81 |
| RESP | CANCER | 0.86 |
| so2_9_clus_0.0 | so2_9_clus_2.0 | -0.96 |
| TCOST_IDX | pct_Single_Unit_ACS | -0.88 |
| tree_5_clus_1.0 | tree_5_clus_3.0 | -0.97 |
| ViolentRate1000 | CrimeRate1000 | 0.97 |

**Table 2.1** Highly correlated variable pairs: Selected (L) and removed (R)

##

## Supplement 3: Sensitivity check

|  |  | **Missing** | **Overall** | **2010-2016** | **2017-2019** | **P-Value** |
| --- | --- | --- | --- | --- | --- | --- |
|  |  |  | 8,208 | 4,471 | 3,737 |  |
| Race/ethnicity, n (%) | | 0 |  |  |  |  |
|  | Hispanic |  | 398 (4.8) | 196 (4.4) | 202 (5.4) |  |
|  | *Non-Hispanic:* | |  |  |  |  |
|  | American Indian or Alaska Native | | – | – | – | 0.104 |
|  | Asian/Mideast Indian | | 59 (0.7) | 36 (0.8) | 23 (0.6) |  |
|  | Black/African-American | | 7,358 (89.6) | 4,027 (90.1) | 3,331 (89.1) |  |
|  | Native Hawaiian/Other Pacific Islander | | – | – | – |  |
|  | White |  | 240 (2.9) | 137 (3.1) | 103 (2.8) |  |
|  | More than one Race | | 78 (1.0) | 34 (0.8) | 44 (1.2) |  |
|  | Unknown |  | 70 (0.9) | 37 (0.8) | 33 (0.9) |  |
| Gender, n (%) | | 0 |  |  |  |  |
|  | Female |  | 3,470 (42.3) | 1,879 (42.0) | 1,591 (42.6) | 0.633 |
|  | Male |  | 4,738 (57.7) | 2,592 (58.0) | 2,146 (57.4) |  |
| Age, mean (SD) | | 0 | 7.0 (4.9) | 7.0 (4.9) | 6.9 (5.0) | 0.312 |
| Insurance, n (%) | | 189 |  |  |  | <0.001 |
|  | Private |  | 1,593 (19.9) | 1,038 (23.3) | 555 (15.6) |  |
|  | Private with Medicaid | | 55 (0.7) | – | 51 (1.4) |  |
|  | Public: Medicaid or Medicare | | 6,238 (77.8) | 3,279 (73.6) | 2,959 (83.0) |  |
|  | Self pay |  | 116 (1.4) | 116 (2.6) | 0 |  |
|  | Misc |  | 17 (0.2) | 17 (0.4) | 0 |  |
| Phenotypes, n (%) | |  |  |  |  |  |
|  | T2-High | 7,739 | 220 (47%) | 192 (57%) | 57 (44%) | 0.0130 |
|  | Atopic | 1,355 | 3,168 (70%) | 1,599 (49%) | 1,569 (44%) | 0.0002 |
|  | Obesity | 7,468 | 175 (24%) | -- | 173 (24%) | n/a |

**Table 3.1:** All asthma visits 2010-2019. An electronic health record (EHR) sensitivity check for a change in the year occurring between the years 2010-2016 and 2017 and 2019. Counts between 1 and 10 are suppressed with “--”.

## Supplement 4: Correlation matrix


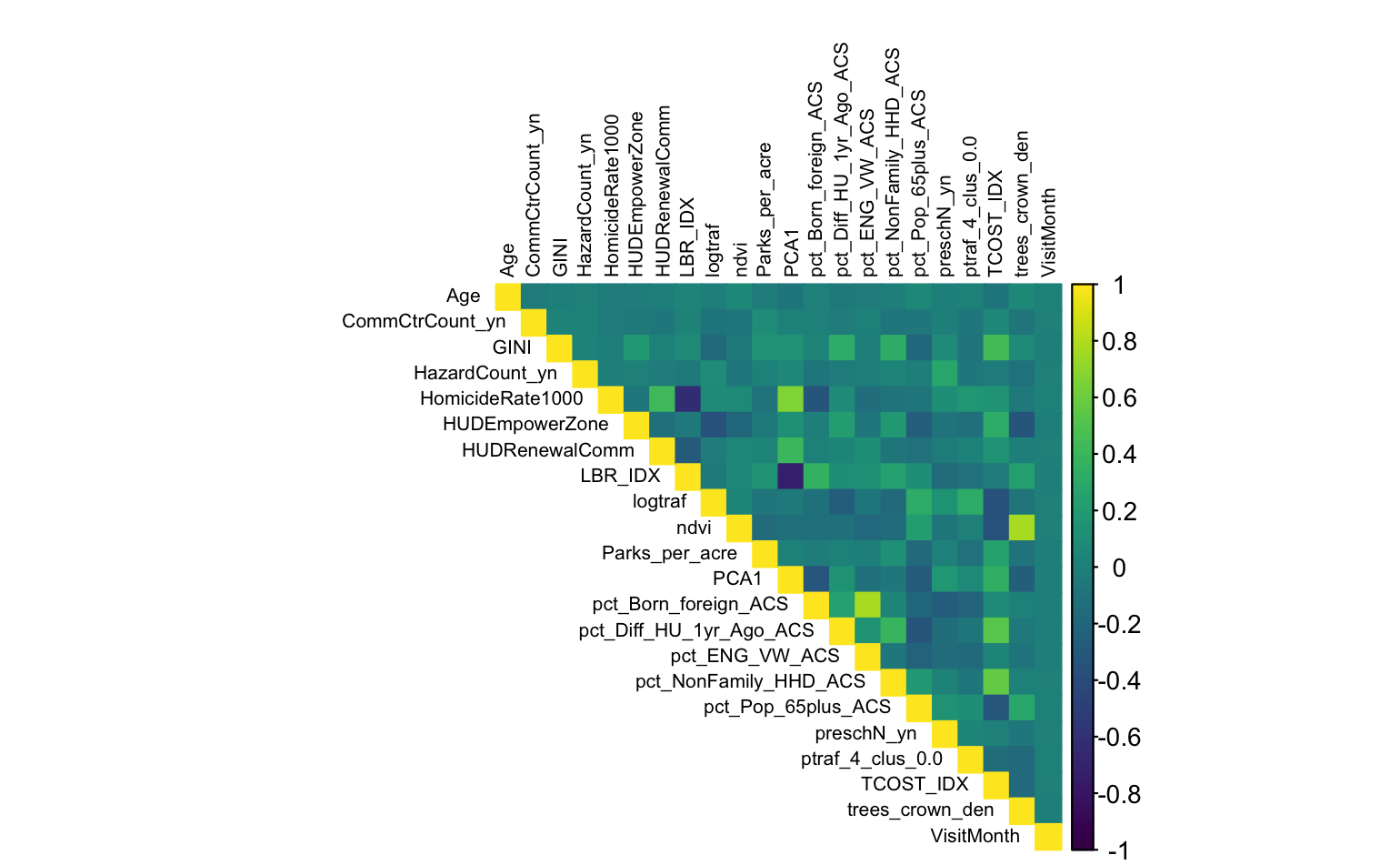


**Figure 4.1** Correlation matrix: Remaining correlations among variables in the final model
